# IUC24429-86 Real-world use of bone-modifying agents (BMA) in patients with metastatic castration-resistant prostate cancer (mCRPC) in the United States

**DOI:** 10.1093/oncolo/oyaf248.048

**Published:** 2025-08-29

**Authors:** Z I Ozay, Y Jo, M Ostrowski, N Sayegh, R Graf, S Roy, E Antonarakis, R McKay, N Agarwal, U Swami

**Affiliations:** Huntsman Cancer Institute, United States; Huntsman Cancer Institute, United States; Huntsman Cancer Institute, United States; Huntsman Cancer Institute, United States; Foundation Medicine, United States; Rush MD Anderson Cancer Center, United States; University of Minnesota, United States; UC San Diego, United States; Huntsman Cancer Institute, United States; Huntsman Cancer Institute, United States

## Abstract

**Background:**

Most patients with prostate cancer experience symptomatic skeletal events, leading to increased morbidity and mortality. BMAs have been approved since 2007 and recommended in mCRPC to prevent SSEs in patients with bone metastases or high-risk of osteoporotic fracture. Herein, we sought to assess real-world trends of BMA administration in patients with mCRPC.

**Methods:**

We used the de-identified nationwide Flatiron Health electronic health record-derived database to extract patient-level data. Eligibility: patients with mCRPC with available date of mCRPC diagnosis and information on receipt of BMA. Patients were categorized into 2 cohorts based on BMA receipt. Treatment trends of BMA by year of mCRPC diagnosis were summarized using frequency and percentages. Baseline characteristics at mCRPC diagnosis (age, race-ethnicity, practice type, insurance) were compared using the Wilcoxon rank-sum and chi-squared tests.

**Results:**

Of 24,105 patients with metastatic prostate cancer, 14,112 with mCRPC were eligible and included. A total of 7,990 (56.6%) received BMA while 6,122 (43.4%) did not. BMA receipt cohort: median age was 75 (IQR 67-81), the majority were White (61%), treated in community practice (87%) and had a commercial health plan (80%). BMA non-receipt cohort: median age was 75 (IQR 68-82), the majority were White (61%), treated in community practice (78%), and had a commercial health plan (76%). Use of BMA decreased over time from 56% in 2013 to 46% in 2024 (Table 1).

**Conclusions:**

Despite recommendations to use BMAs in patients with mCRPC and bone metastasis or high-risk of fracture, our findings reveal a low utilization rate of BMAs in mCRPC with a notable decline over time. This highlights the need to incorporate BMAs in clinical practice.

**Table 1:** Trends of BMA usage over time (2013-2024).

Year, *n* (%)	2013 *N* = 549	2014 *N* = 1035	2015 *N* = 1223	2016 *N* = 1345	2017 *N* = 1441	2018 *N* = 1346	2019 *N* = 1469	2020 *N* = 1380	2021 *N* = 1352	2022 *N* = 1393	2023 *N* = 1196	2024 *N* = 383
BMA receipt	316 (56%)	674 (65%)	792 (65%)	837 (62%)	886 (61%)	766 (57%)	816 (56%)	766 (56%)	692 (51%)	713 (51%)	556 (46%)	176 (46%)
Denosumab	190 (60.1%)	450 (66.8%)	557 (70.3%)	599 (71.6%)	606 (68.4%)	533 (70%)	526 (64.5%)	488 (63.7%)	410 (59.2%)	414 (58.1%)	290 (52.2%)	94 (53%)
Zoledronic acid	120 (38%)	220 (32.6%)	228 (28.7%)	233 (27.8%)	270 (30.5%)	225 (29%)	281 (34.4%)	273 (35.6%)	277 (40%)	295 (41.4%)	258 (46.4%)	82 (47%)
Teriparatide/other bisph.	6 (1.9%)	4 (0.6%)	7 (1%)	5 (0.6%)	10 (1.1%)	8 (1%)	9 (1.1%)	5 (0.7%)	5 (0.7%)	4 (0.5%)	8 (1.4%)	0

